# Mapping malaria risk and vulnerability in the United Republic of Tanzania: a spatial explicit model

**DOI:** 10.1186/s12963-015-0036-2

**Published:** 2015-02-03

**Authors:** Michael Hagenlocher, Marcia C Castro

**Affiliations:** Interfaculty Department of Geoinformatics – Z_GIS, University of Salzburg, Schillerstr. 30, 5020 Salzburg, Austria; Department of Global Health and Population, Harvard School of Public Health, 677 Huntington Avenue, Boston, MA 02115 USA

**Keywords:** Malaria, Risk, Vulnerability, Spatial composite indicators, Tanzania

## Abstract

**Background:**

Outbreaks of vector-borne diseases (VBDs) impose a heavy burden on vulnerable populations. Despite recent progress in eradication and control, malaria remains the most prevalent VBD. Integrative approaches that take into account environmental, socioeconomic, demographic, biological, cultural, and political factors contributing to malaria risk and vulnerability are needed to effectively reduce malaria burden. Although the focus on malaria risk has increasingly gained ground, little emphasis has been given to develop quantitative methods for assessing malaria risk including malaria vulnerability in a spatial explicit manner.

**Methods:**

Building on a conceptual risk and vulnerability framework, we propose a spatial explicit approach for modeling relative levels of malaria risk - as a function of hazard, exposure, and vulnerability - in the United Republic of Tanzania. A logistic regression model was employed to identify a final set of risk factors and their contribution to malaria endemicity based on multidisciplinary geospatial information. We utilized a Geographic Information System for the construction and visualization of a malaria vulnerability index and its integration into a spatially explicit malaria risk map.

**Results:**

The spatial pattern of malaria risk was very heterogeneous across the country. Malaria risk was higher in Mainland areas than in Zanzibar, which is a result of differences in both malaria entomological inoculation rate and prevailing vulnerabilities. Areas of high malaria risk were identified in the southeastern part of the country, as well as in two distinct “hotspots” in the northwestern part of the country bordering Lake Victoria, while concentrations of high malaria vulnerability seem to occur in the northwestern, western, and southeastern parts of the mainland. Results were visualized using both 10×10 km^2^ grids and subnational administrative units.

**Conclusions:**

The presented approach makes an important contribution toward a decision support tool. By decomposing malaria risk into its components, the approach offers evidence on which factors could be targeted for reducing malaria risk and vulnerability to the disease. Ultimately, results offer relevant information for place-based intervention planning and more effective spatial allocation of resources.

**Electronic supplementary material:**

The online version of this article (doi:10.1186/s12963-015-0036-2) contains supplementary material, which is available to authorized users.

## Background

Despite recent progress in reducing malaria morbidity and mortality as a result of the expansion and intensification of malaria control programs, approximately half of the world remains at risk of contracting the disease [1], and in 2012 about 207 million cases and 627,000 malaria-related deaths were observed [2]. Currently, most strategies for malaria prevention and control tend to concentrate on reducing exposure to mosquitos or treating infections [2,3]. While these strategies do provide tangible health benefits, integrative approaches (accounting for socioeconomic, cultural, behavioral, environmental, and political aspects) that also aim at reducing individual vulnerabilities to the disease are needed as countries achieve very low levels of transmission [4-6].

In recent years, a range of malaria risk assessments has been carried out, focusing on (i) malaria transmission risk [7-11], (ii) malaria risk factors [12-14], (iii) the links between climate change and malaria risk [15-17], and (iv) spatial population datasets for mapping populations at risk [18-20].

Malaria risk assessments focus on the probability of harmful consequences resulting from interactions between the hazard (or the disease; measured by the entomological inoculation rate [EIR]) and the vulnerability of the exposed population; thus, they are commonly carried out once the hazard is prevalent. Vulnerability assessments focus on identifying and analyzing a wide range of socioeconomic, demographic, biological, cultural, and governance-related factors that can potentially impact human susceptibility and lack of resilience to the disease. Therefore, mapping vulnerability and integrating the results in a spatial explicit risk assessment can provide evidence for planning preventative interventions, by targeting factors that can reduce susceptibility and increase resilience. Although the focus on malaria risk and vulnerability is increasingly gaining ground in the malaria literature [21], little emphasis has been given to integrating spatial explicit information on prevailing vulnerabilities into malaria risk assessments.

This paper addresses these issues, and aims to model malaria vulnerability in the United Republic of Tanzania using a spatial explicit approach and combine it with existing hazard/disease information (i.e., EIR) to produce a malaria risk map.

## Methods

### Study area

**Figure 1:**
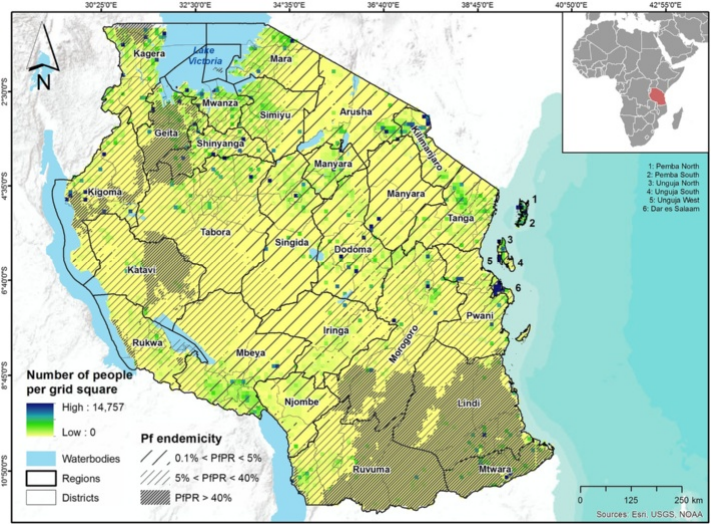
**United Republic of Tanzania, detailed by regions and districts.** The map shows the number of people per 10×10 km^2^ grid square, as well as the spatial distribution of *Plasmodium falciparum (Pf)* malaria stratified by endemicity class (as provided by the Malaria Atlas Project [1]).

The United Republic of Tanzania (from now on referred to as Tanzania) is located in eastern Africa between latitude 1° and 12° South and longitude 29° and 40° East, with an area of approximately 945,203 km^2^, including bodies of water. Administratively, the country is divided into 30 regions: 25 in Tanzania Mainland and five in Zanzibar, the group of islands off the eastern coast of Tanzania Mainland (Figure 1).

According to the 2012 Population and Housing Census [22], Tanzania has a population of 44.9 million inhabitants, of which 43.6 million live in Tanzania Mainland and 1.3 million in Zanzibar. The majority of the country is sparsely populated (population density of 51 persons/km^2^), with the exception of Dar es Salaam (3,133 persons/km^2^) and Ugunja West (2,581 persons/km^2^). The current growth rate is 2.7%, and 28.3% of the population live in urban areas [22].

The country has a tropical climate that varies regionally, due to topography. With the exception of a narrow coastal strip, most of the country belongs to the East African highlands. The coastal regions are warm and humid, while the highland areas are more temperate. Driven by the movement of the Inter‐Tropical Convergence Zone (ITCZ), the country reveals two bimodal rainfall patterns: the north and the east experience two distinct wet periods – the short rains in October to December and the long rains in March to May, whilst the southern, western, and central parts of the country have one wet season that happens in October to April or May.

According to the 2013 World Malaria Report [2], Tanzania is among the six countries with the highest malaria burden in the World Health Organization (WHO) African region. Although malaria is largely under control in Zanzibar, it is still a major public health problem on the mainland. Despite recent progress in reducing the burden of the disease, malaria was the second major cause of disease burden in 2010, as measured by disability-adjusted life years (DALYs). In terms of the number of years of life lost (YLL), HIV/AIDS (21%), malaria (14%), and lower respiratory infections (9%) were the top-three-ranking causes in the country in 2010 [23].

According to the President’s Malaria Initiative (PMI), stable malaria transmission with seasonal variation occurs in approximately 20% of the country, while unstable seasonal malaria occurs in another 20%. The remaining endemic areas are characterized as stable perennial transmission [24]. As a consequence, approximately 93% of the mainland population lives in areas where malaria is transmitted, while the entire population of Zanzibar is prone to malaria infection [24]. Although the promotion and distribution of (long-lasting) insecticide-treated nets (LLINs/ITNs), the implementation of indoor residual spraying (IRS), and the scale-up of both artemisinin-based combination therapy (ACT) and intermittent preventive treatment in pregnancy (IPTp) have substantially reduced morbidity and mortality in the past decade, there are still approximately 60,000 to 80,000 malaria-attributable deaths every year [25,26]. The most vulnerable groups (e.g., the poor, children under 5, pregnant women) carry the highest burden [3,26-28].

### Modeling framework

**Figure 2:**
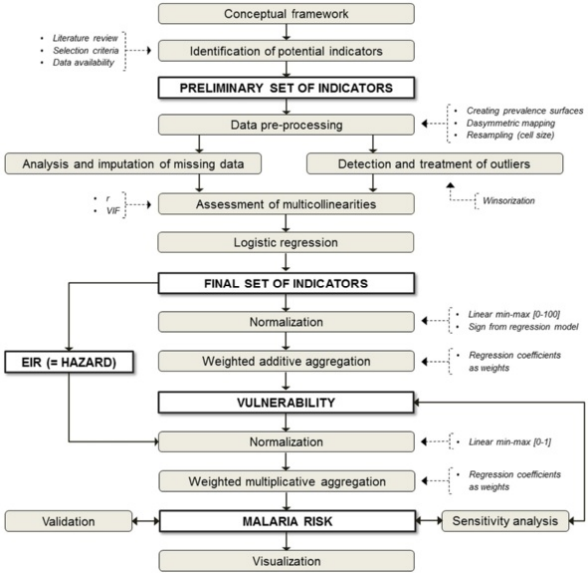
**Modeling framework.** Includes all stages of the modeling process, from conceptualization to visualization. Grey boxes indicate modeling phases; white boxes represent input/output layers.

For the construction of the spatial risk and vulnerability surfaces an iterative modeling framework, pursuing a traditional Multi-Criteria Assessment (MCA) approach [29], was applied following guidelines proposed by the Organization for Economic Co-operation and Development [30]. The modeling process is displayed in Figure 2. Relevant modeling phases include: (1) definition of the conceptual risk and vulnerability framework; (2) identification of potential hazard, vulnerability, and exposure indicators based on a systematic review of literature, selection criteria, and data availability; (3) data preprocessing; (4) analysis and imputation of missing data; (5) outlier detection and treatment; (6) assessment and reduction of existing multicollinearities; (7) normalization; (8) logistic regression analysis for evaluating the relationship between potential explanatory risk factors (identified in phase two) and malaria endemicity; (9) weighting and aggregation of indicators and domains in a vulnerability index; (10) aggregation of hazard (EIR) and vulnerability in a malaria risk map; (11) sensitivity analysis; (12) validation; and (13) visualization of modeling results.

A spatial explicit model that builds on gridded datasets was selected to reflect the spatial distribution of malaria risk and vulnerability independent of a-priori defined boundaries, such as administrative units. We used both regions and districts as additional reporting units, since the National Malaria Control Program (NMCP) spatially targets some interventions (e.g., IRS) at this level. Thus, results can provide evidence for local policymaking and intervention planning.

### Conceptual framework

As no universal definition exists for risk and vulnerability (yet) [3,31], it is essential to choose an approach that is appropriate for the context in which the risk and vulnerability assessments are embedded [32] and considering the hazard that is addressed (e.g., climate change, natural hazards, vector-borne diseases, etc.). Conceptual frameworks are valuable tools for the operationalization of the multifaceted nature of risk and vulnerability, as they guide the development of appropriate models as well as the systematic identification of indicators [33].

**Figure 3:**
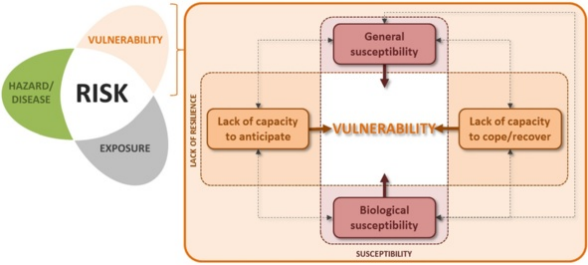
**Conceptual risk and vulnerability framework.** Risk is defined as a function of hazard (here proxied by the EIR) and the vulnerability of exposed population groups (adapted from [34,35]).

In the context of vector-borne diseases like malaria, risk is a function of the hazard (e.g., represented by the entomological inoculation rate - EIR) and the vulnerability of exposed population groups (Figure 3).

The interrelation of these three components (hazard, vulnerability, and exposure) reflects the vital importance of feedback when dealing with the malaria transmission cycle. For example, ITN usage or IRS minimizes vulnerability, but by also reducing vector populations they can fundamentally affect transmission and thus impact both the hazard/disease and exposure components of the framework. Within this context, we define vulnerability (VU) as the predisposition of the society and its population to the burden of malaria, taking into account spatial differences in their susceptibility and lack of resilience. Thereby, susceptibility represents the propensity of individuals to be negatively affected by malaria as a result of both generic and biological susceptibility factors, while lack of resilience refers to the inability to respond and absorb negative impacts as a result of lacking capacity to anticipate, cope with, or recover from the (burden of the) disease [34,35]. In this paper we consider generic susceptibility (SUS), biological susceptibility (BIO), and the lacking capacities to anticipate (C2A) and to cope (C2C) as different domains of vulnerability (VU):

$$ VU=f\;\left(SUS,BIO,C2A,C2C\right) $$

Each domain is composed by different factors: socioeconomic (e.g., education, knowledge, behavior, occupation, income, livelihoods, etc.), biological (e.g., age, acquired immunity, drug resistance, nutritional/health status), and institutional (e.g., health services and policy, including access to health care, quality of care, malaria control strategies, etc.).

By decomposing the complex and multidimensional nature of malaria risk into its different components (i.e., hazard, exposure, vulnerability), and vulnerability into its different domains and factors, the framework guides the conceptualization and operationalization of malaria risk and vulnerability in a spatial explicit model.

### Data sources

Guided by the conceptual framework and based on a systematic review of literature and available data, a preliminary set of 20 socioeconomic, demographic, environmental, and governance-related vulnerability indicators, as well as one hazard/disease-related indicator, were identified (see Table 1).

**Table 1:** **Malaria risk factors, resolution, reference year, expected relationship with malaria (sign, weight), and data source**

**Indicator name**	**Resolution**	**Date**	**Sign** ^**a**^	**Weights** ^**b**^	**Data source**
*Hazard/disease-related (HAZ) indicators*					
HAZ_01: Entomological inoculation rate (EIR)		2010	+	0.476	Malaria Atlas Project
*General susceptibility (SUS) indicators*					
SUS_01: Agricultural areas (%)	300 m	2009	+	0.023	ESA GlobCover
SUS_02: Density of violent conflicts (km^2^)	Point layer	1997-2012	+	- ^c^	ACLED
SUS_03: Location of refugee camps	Point layer	2013	-	0.003	UNHCR
SUS_04: Poor housing conditions (%)	Point layer	2011/12	-	0.022	THMIS
SUS_05: Occupation: forestry/agriculture/fisheries (%)	Point layer	2011/12	-	0.019	THMIS
SUS_06: Rural extent^c^	1 km	2002	-	- ^c^	MODIS
SUS_07: Water bodies (%)	300 m	2009	+	0.020	ESA GlobCover
*Biological susceptibility (BIO) indicators*					
BIO_01: Children under the age of 5 (%)	1 km	2010	-	0.005	WordPop
BIO_05: Women of childbearing age (%)	1 km	2010	+	0.005	WorldPop
BIO_06: Number of HIV infected individuals (15–49 years)	Polygon layer	2007	-	0.054	UNAIDS
BIO_08: Number of stunting children under 5 years	Polygon layer	2007	+	0.020	FAO
*Lack of capacity to anticipate (C2A) indicators*					
C2A_01: No/primary education (%)	Point layer	2011/12	+	0.038	THMIS
C2A_02: Does not know how to avoid malaria (%)^c^	Point layer	2011/12	+	- ^c^	THMIS
C2A_03: No phones (cell/landline) (%)	Point layer	2011/12	+	0.062	THMIS
C2A_04: Child did not sleep under net last night (%)	Point layer	2011/12	-	0.066	THMIS
C2A_05: No indoor residual spraying (%)	Point layer	2011/12	-	0.028	THMIS
*Lack of capacity to cope (C2C) indicators*					
C2C_01: Travel time to closest urban center (hours)	1 km	2000	+	0.018	JRC
C2C_02: No health insurance (%)	Point layer	2011/12	-	0.001	THMIS
C2C_03: No bicycle/motorcycle/car or truck (%)	Point layer	2011/12	-	0.083	THMIS
C2C_04: Density of health-related projects (km^2^)	Point layer	2011/12	-	0.057	World Bank

^a^Sign indicates if high indicator values increase (+) or decrease (−) risk. The sign is derived from the regression analysis. ^b^Weights are derived from the coefficients of the regression analysis. ^c^These indicators were removed from the analysis as they were not statistically significantly (p-value < 0.05) related to malaria endemicity in the study area.

These indicators were extracted from several data sources, as shown in Table 1. The 2011–12 HIV/AIDS and Malaria Indicator Survey (THMIS) [26] provided the following information (all variables are binary): (i) poor housing conditions, based on rudimentary or natural floor, wall, and ceiling material [27,13]; (ii) occupation of the respondent was agriculture, fishing, or forestry [3,27,36,37]; (iii) primary education or less [3,38]; (iv) lacking knowledge on how to avoid malaria [38]; (v) lacking ownership of cell phones or landlines [27], used as a proxy for access to information; (vi) child did not use an ITN/LLIN the night before the interview [3,14,27,37]; (vii) house had not been sprayed in the previous 12 months [13,27]; (viii) lack of any health insurance [12]; and (ix) lack of physical household assets in regards to mobility (bicycle/motorcycle/car/truck) [12,39]. Following a workflow published by Lamarange et al. [40], we created continuous prevalence surfaces from DHS data using a kernel estimator approach. This was done in R statistical software [41] using the prevR package.

WorldPop (formerly known as AfriPop) provides gridded population data at a resolution of 1 km [42], and the following information was obtained: (i) women of childbearing age, here considered as 15 to 49 years old [3,13,27,43] and (ii) children under the age of 5 [3,14,27,28,43]. Both variables were standardized by the total population, utilizing a total population grid also available at WorldPop.

The prevalence of stunting among children under 5 [3,13,37,43] by the district level was downloaded from the Food and Agriculture Organization (FAO; http://www.fao.org/geonetwork/srv/en/main.home). Subnational HIV prevalence [3,13] for people aged 15 to 49 (reference year 2007) was downloaded from the Joint United Nations Programme on HIV/AIDS (UNAIDS; http://www.unaids.org/en/regionscountries/countries/unitedrepublicoftanzania/). To convert these two prevalence datasets into continuous surfaces we used a dasymetric mapping approach [44]. All analyses were done in ESRI ArcGIS 10.1 (ESRI, Redlands, CA).

To account for the impacts of the local environment on human malaria susceptibility [27,36,45], the 2009 ESA GlobCover land use/land cover (LULC) product (300 m resolution) was acquired (www.edenextdata.com). Based on the LULC dataset two additional surfaces were created: (i) relative share of water bodies and (ii) relative share of agricultural areas (i.e., irrigated crop land, rainfed crop land, closed to open broad-leaved forest regularly flooded, closed broad-leaved forest permanently flooded, closed to open vegetation regularly flooded).

Africa-wide data on political conflict from 1997 to 2012 are available through the Armed Conflict Location and Event Dataset (ACLED; http://www.acleddata.com/) and were used to obtain a surface of conflict density per km^2^. Two types of events that might have an impact on health care systems, including access to health care, were taken into account [36]: (i) battle, including cases when no territory was exchanged, rebels won territory, and government regained territory; and (ii) violence against civilians. Nonviolent protests and riots were not included, since they can be interpreted as proxies for political empowerment and participation.

Information on the current location of refugee settlements [46,47] was obtained from the United Nations High Commissioner for Refugees (UNHCR; http://www.unhcr.org/pages/49e45c736.html). A global map of accessibility (http://bioval.jrc.ec.europa.eu/products/gam/index.htm) that shows the travel time in hours to urban centers was gathered from the Joint Research Center (JRC) of the European Commission (EC), and used as a proxy for access to health facilities [27,36,46]. Donor aid for health-related projects [46,48] was acquired from the World Bank (http://data.worldbank.org/country/tanzania). We obtained a grid with rural extents [25,27,36] from Moderate Resolution Imaging Spectroradiometer (MODIS) 500-m satellite data [49,50]. Moreover, a gridded surface showing the spatial distribution of *Plasmodium falciparum* entomological inoculation rate (EIR) was downloaded from the Malaria Atlas Project website [1] as a hazard/disease indicator. Lastly, a layer representing estimated levels of *Plasmodium falciparum* malaria endemicity was acquired from the same website and used as an input (dependent variable) for the regression model, as well as to validate the final malaria risk surface.

### Data preprocessing

All surfaces were resampled to the same cell size (10×10 km^2^) and cropped to the boundaries of the study area. As malaria risk and vulnerability are human-centered concepts [48], areas covered by major water bodies were not included in the analysis.

We used descriptive statistics and box plots to evaluate the degree of missing data as well as potential outliers. While no major problems were observed regarding missingness, outliers were found in three indicators (number of HIV infected individuals, number of stunting children under 5, and population density) and treated using a winsorization approach [30]. To assess if multicollinearity was an issue among the variables comprising each domain, we calculated the Pearson correlation coefficient (*r*) and the variance inflation factor (Additional file 1: Tables S1-S8). We did not observe high correlations (r > 0.9) or a high VIF value (VIF > 5). Consequently, all variables were included in the regression analysis. To render the variables comparable, all indicators were standardized (*v*_*i*_*′*) to a value ranging from 0 to 100 using linear min-max normalization defined as:

$$ {v}_i^{\prime }=\frac{\left({v}_i-{v}_{\min}\right)}{\left({v}_{\max }-{v}_{\min}\right)}\ast 100 $$

where *v*_*i*_ refers to the observed value of the variable in pixel *i*, and *v*_*min*_ and *v*_*max*_ represent the minimum and maximum values, respectively, of the variable. Detailed maps showing the spatial patterns of all indicators utilized in this study are presented of the Supplemental Material (see Additional file 1: Figure S1).

### Correlates of endemicity

Logistic regression has been widely used in epidemiological studies to identify relevant correlates of disease [14,51-54]. Since our model is spatially explicit, and all information is represented by grids, we applied logistic regression using the pixel as the unit of analysis to appraise the relationship between malaria endemicity (measured as the proportion of people infected in the pixel, as reported by the Malaria Atlas Project), and a set of 21 social, demographic, economic, environmental, biological, institutional, and disease-related variables (Table 1). A total of 10,476 pixels (10×10 km of spatial resolution) were available for the regression analysis, and the model is represented by:

$$ \ln \left(\frac{P_i}{1-{P}_i}\right)={\beta}_0+{\beta}_1{x}_{1,i}+\cdot \cdot \cdot +{\beta}_n{x}_{n,i} $$

where *P*_*i*_ is the expected value of the dependent variable *y*_*i*_ at pixel *i* (i.e., the likelihood of malaria prevalence), *x* refers to the *n* independent variables (i.e., the potential risk factors), and *β* are the estimated regression coefficients.

The fit of the model was evaluated using several tests, including the likelihood ratio test (LRT), an assessment of the prediction accuracy, and McFadden’s pseudo R-squared. The LRT was performed by comparing the proposed model with a restricted model where the explanatory variables of interest were omitted, while the p-values of the test were calculated using the chi-square distribution. The prediction accuracy of the model was evaluated by splitting the data into two subsets using random sampling: (i) a training dataset with 75% of the observations (n = 7,834) and (ii) a testing dataset with the remaining 25% of the observations (n = 2,633). The training data were used to predict malaria endemicity for the testing data, and for ease of interpretation, both predicted and observed endemicity values were categorized as low (0.0-0.33), medium (0.33-0.66), and high (0.66-1.0). All statistical analyses were performed in R statistical software using the glm command [41].

### Composite vulnerability index

Standardized values (*v*_*i*_*′*) were used to produce a surface representing each of the four domains of vulnerability previously described (SUS, BIO, C2A, C2C), considering the weighted sum of the variables in each domain and using the coefficients from the regression analysis (Table 1) as weights. The vulnerability index (*VU*) was then obtained as the weighted sum of all vulnerability indicators according to the following equation: $VU={\displaystyle {\sum}_{i=1}^n{w}_iv{\prime}_i}$, where *n* equals the number of vulnerability indicators, and *w*_*i*_ are the weights for each normalized dataset *v*_*i*_*′*; these weights were obtained from the coefficients of the logistic regression results and were normalized to add up to 1. The final *VU* surface was normalized within a range from 0 to 1, where 0 indicates no malaria vulnerability, and 1 represents the highest vulnerability.

### Mapping malaria risk

We used EIR estimates as a proxy for malaria hazard. The EIR layer was normalized to a range from 0 to 1 and then combined with the normalized *VU* surface using a multiplicative weighted aggregation, *RISK* = ((*w* ∗ *EIR*) ∗ ((1 − *w*) ∗ *VU*), where the weight for the EIR surface was obtained from the logistic regression analysis (Table 1). A multiplicative approach to aggregation was chosen to ensure that risk is 0 if one of the two components (i.e., vulnerability or hazard/exposure) is 0. For ease of interpretation the final risk surface was standardized within a range from 0 to 1, where 0 reflects no malaria risk, and 1 represents the highest risk. We did not include an explicit exposure variable since the number of people is already indirectly included in the other components of risk.

### Sensitivity analysis

We assessed the sensitivity of the modeled risk and vulnerability surfaces to the choice of indicator weights. We conducted a sensitivity analysis, which targets one index construction stage at a time (here: weighting), while all other stages (normalization, aggregation, etc.) are held constant [55-57]. In addition to using standardized regression coefficients as indicator weights (Table 1), we thus modeled malaria risk and vulnerability using weights based on principal component analysis (PCA) [30] and equal weights (see Additional file 1: Table S9). Following a workflow proposed by Hagenlocher et al. [58], risk and vulnerability surfaces obtained from these two additional weighting schemes were compared with those obtained utilizing weights based on the logistic regression (see Additional file 1: Figure S3). Three assessments were used: (i) the absolute difference between the pixel values of the surfaces; (ii) the Pearson correlation coefficient between the surfaces; and (iii) the local Moran’s I statistic [59] applied to the absolute differences obtained in (i) in order to identify areas where the differences were significantly large or small [58].

## Results

### Correlates of malaria

Multivariate logistic regression analysis suggested that 18 out of 21 factors were significantly (p-value < 0.05) related to *Pf* endemicity in the study area (Table 1).

No significant association was found between *Pf* endemicity and conflict density, knowledge on how to avoid malaria, and rural extent. Some variables showed unexpected relationships with malaria endemicity (e.g., IRS, bed net usage by a child, children under the age of 5, and poor housing conditions), although this has been reported previously by other studies. For example, a negative association between malaria prevalence and no usage of bed nets was also observed in Tanzania [60]. In the case of IRS, this intervention has been implemented only in some districts bordering Lake Victoria and in Zanzibar. Thus, the potential impact is highly localized and may not be captured in a regression model that assumes that relationships are stationary across space. The model had a good fit, as indicated by a highly significant LRT statistic (p < 0.01), and an R Square of 0.76.

### Malaria risk and vulnerability

**Figure 4:**
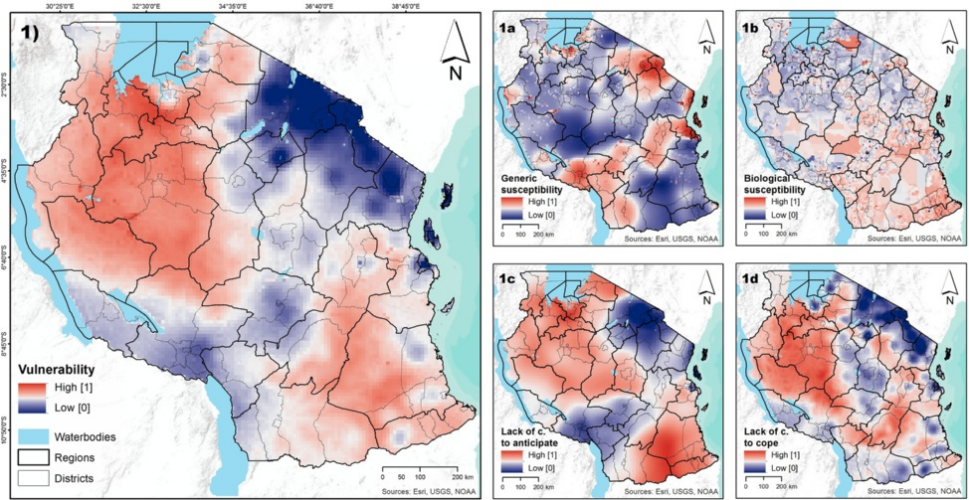
**Modeled surfaces of vulnerability to malaria, including the four vulnerability domains. Panel 1** shows the vulnerability to malaria. **Panels 1a** to **1d** show the four domains of vulnerability: generic susceptibility (SUS), biological susceptibility (BIO), lack of capacity to anticipate (C2A), and lack of capacity to cope (C2C). All surfaces have a 10 km spatial resolution.

Figure 4 shows the *VU* surface calculated for Tanzania, as well as each of its four domains (SUS, BIO, C2A, and C2C), using a 10×10 km grid; administrative boundaries – regions and districts – are shown for reference. Grid cells of relative high levels of vulnerability are displayed in shades of red, while those of low vulnerability are displayed in shades of blue. Malaria vulnerability is generally higher in Mainland areas (mean = 0.66, in a scale from 0 to 1) compared to Zanzibar (mean = 0.37). Concentrations of high malaria vulnerability seem to occur in the northwestern, western, and southeastern parts of the Mainland (Figure 4, panel 1).

By showing the four domains that compose vulnerability it is possible to observe that high vulnerability does not necessarily imply that all four domains are high. For example, areas in the northwestern part of the country reveal relatively high levels of vulnerability (Figure 4, panel 1), a result of low capacity to anticipate or cope with the disease (Figure 4, panels 1c and 1d), despite the relatively low generic and biological susceptibility to malaria (Figure 4, panels 1a and 1b). This has important implications for selecting and targeting strategies to reduce vulnerability to malaria.

**Figure 5:**
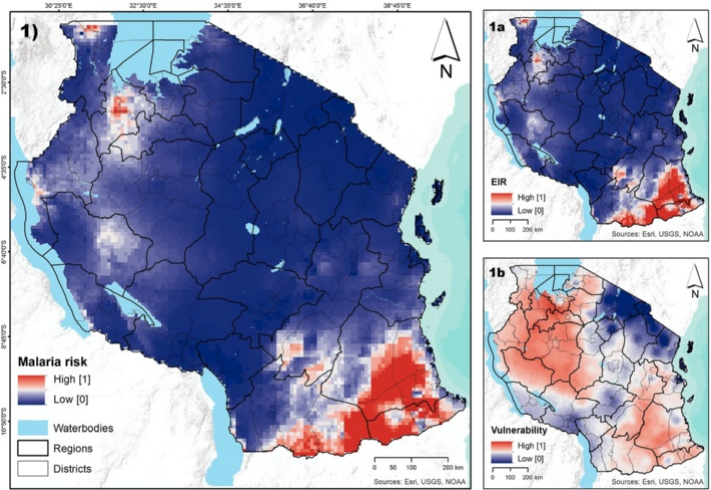
**Modeled surfaces of malaria risk, including the EIR and malaria vulnerability. Panel 1** shows prevailing levels of malaria risk. **Panels 1a** and **1b** show the two components of malaria risk: entomological inoculation rate (EIR) and vulnerability. All surfaces have a 10 km spatial resolution.

Figure 5 shows the computed malaria risk for Tanzania (Panel 1), as well as its two components: hazard (Panel 1a) and vulnerability of exposed population groups (Panel 1b). There is a notable similarity between the risk (Panel 1) and hazard (Panel 1a) surfaces, a result of the large coefficient for EIR that resulted from the logistic regression model (Table 1).

Thus, although malaria vulnerability is relatively high for most parts of the Mainland (with the exception of the northeastern parts bordering Kenya, and a small area to the north of Lake Malawi), malaria risk is relatively low for large parts of the country as low levels of EIR prevail. High levels of malaria risk concentrate in the southeastern part of the country, as well as in two distinct “hotspots” in the northwestern part of the country, bordering Lake Victoria (Figure 5, panel 1). Malaria risk is higher in Mainland areas (mean = 0.09, in a scale from 0 to 1) compared to Zanzibar (mean = 0.002), as a result of very low levels of both *Pf* malaria EIR and malaria vulnerability in Zanzibar.

**Figure 6:**
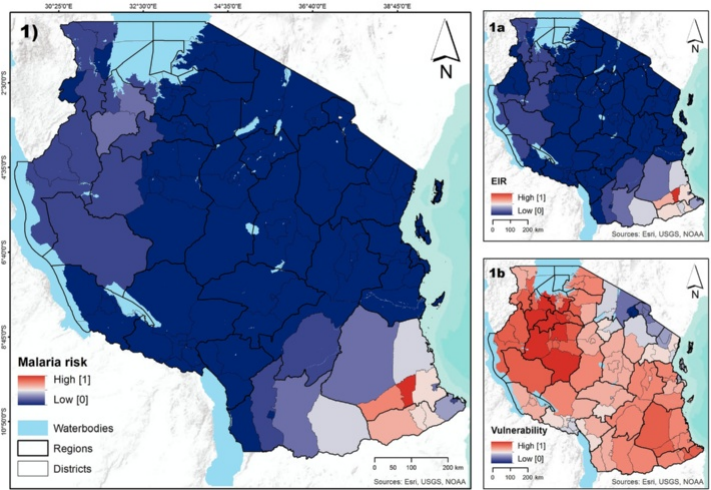
**Modeled surfaces of malaria risk, including the EIR and malaria vulnerability by district. Panel 1** shows prevailing levels of malaria risk. **Panels 1a** to **1b** show the two components of malaria risk: entomological inoculation rate (EIR) and vulnerability, respectively. District values are pixel averages obtained from Figure 5.

**Figure 7:**
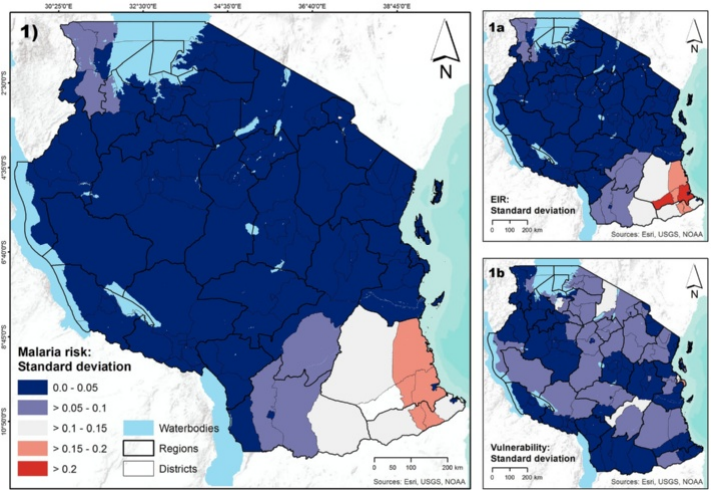
**Standard deviation in district values of malaria risk and its two components. Panel 1** shows the variability in malaria risk. **Panels 1a** and **1b** show the variability in the two components of malaria risk (i.e., EIR and vulnerability).

Figure 6 shows the malaria risk index and its components aggregated at the district level, a resolution often utilized by policymakers. At this aggregation level, the district with highest malaria risk was Ruangwa District in the Lindi Region (score of 1), which presented a high index of vulnerability (0.78) and a very high EIR (score of 1). In contrast, the districts with lowest malaria risk were Micheweni District (score of 0) in Pemba North, Mkoani District (score of 0) in Pemba South, Mbeya Urban District (score of 0, in a scale from 0 to 1) in the Mbeya region, and North A District (0.0, in a scale from 0 to 1) in Unguja North. Although all of them revealed medium vulnerability to the disease (between 0.32 and 0.44), they were classified as no risk areas as EIR was 0. While the aggregation process removes the variability within the district, and thus the maps portray a smoothed scenario, the spatial patterns identified in Figure 5 are also discernible in Figure 6. However, assessing the magnitude of the variability is important. Thus Figure 7 shows the standard deviation of the malaria risk index and its two components within each district. Districts with high variability are displayed in shades of red, and those with low variability in shades of blue.

High standard deviation suggests high variability within the district, and therefore interventions to reduce risk should be targeted spatially, addressing local idiosyncrasies observed in EIR and/or vulnerability. As an example, all the pixels in Arusha District had the same value, and thus no variability was observed for EIR and vulnerability. In Lindi Rural District, however, the pixel values for EIR and vulnerability vary a lot, revealing a high degree of variability within the district.

### Contributions of different factors in each vulnerability domain

**Figure 8:**
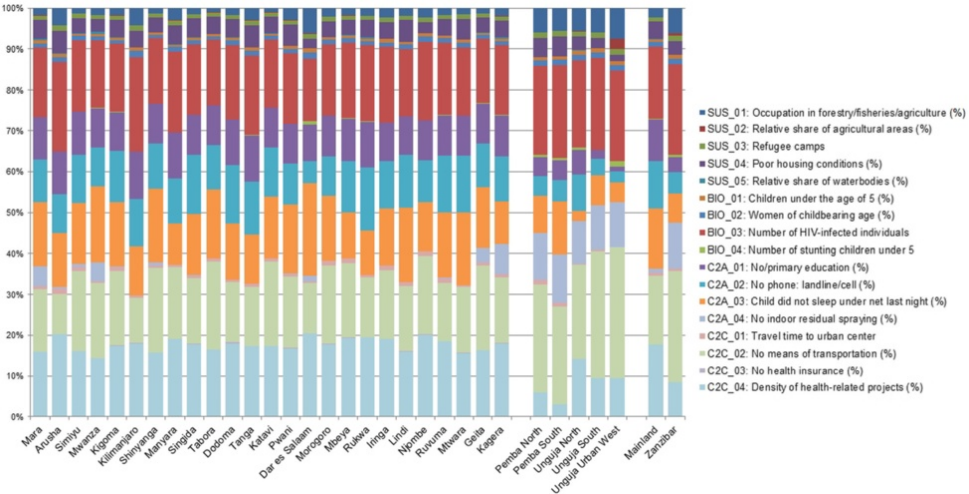
**Relative contribution of vulnerability indicators by region.** Figure 8 shows the relative contribution of the 17 vulnerability indicators for each of the 30 regions.

To better understand which factors contributed the most to the final modeled surface of vulnerability (Figure 4, panel 1), the relative share (in percentages) of individual indicators was calculated by region. Figure 8 shows the contribution of each of the 17 final vulnerability indicators. While some indicators contribute roughly the same across regions (such as the relative share of water bodies), some vary considerably, impacting the observed vulnerability levels only in some regions (such as the number of stunted children under 5). Contrasting Zanzibar and Mainland, important differences are in the contribution of usage of mosquito nets, indoor residual spraying, the lack of education, and whether or not people have a phone. A more detailed comparison of the indicators for Zanzibar and Mainland is presented (see Additional file 1: Figure S2).

### Influence of indicator weights on modeled malaria risk and vulnerability

The outcomes of the sensitivity analysis are shown in (Additional file 1: Figures S3 to S5). Results for malaria vulnerability show that, although the overall picture is similar, the vulnerability surfaces vary especially in the northwestern part of the study area when comparing the different weighting schemes. However, as indicated by the local Moran’s I, for most districts this variation is not significant, which is also confirmed by the strong Pearson correlation coefficient between the different vulnerability surfaces (*r* = 0.74 for the vulnerability surfaces based on weights obtained from the regression and from PCA, and *r* = 0.78 when comparing weights from the regression analysis with equal weights). Despite the minor impact of the weighting scheme on the modeled vulnerability surface, the choice of the weighting scheme has hardly any impact on the risk surface, which is also indicated by the strong positive correlation between the risk surface created using regression coefficients as weights and the risk surface that was created using PCA to obtain indicator weights (*r* = 0.99), and the surface based on equal weights (*r* = 0.99).

### Validation

Cross-validation of the final risk map was carried out against (i) the prevalence of malaria infection as measured by the THMIS, and (ii) the levels of *Plasmodium falciparum* malaria endemicity in the country as reported by the Malaria Atlas Project [1]. As the results of the rapid diagnostic tests (RDTs) provided by the THMIS were available for the 30 regions of the country, all datasets were aggregated to this level, and standardized to a range from 0 to 100. To assess the accuracy of the model we calculated the Pearson correlation and plotted the normalized layers in a scatter plot (see Additional file 1: Figure S4). The analysis revealed a strong relationship between malaria risk and malaria prevalence as measured by the THMIS (*r* = 0.65) in the 30 regions.

## Discussion

Using a logistic regression model we selected 18 indicators to model malaria risk and vulnerability in Tanzania. Our results show that malaria risk and its two components (vulnerability and hazard) varied across the country, but also that areas at risk did not necessarily have adverse conditions in both components. Since gridded maps are of limited use for policymakers, results were summarized at the district level, revealing differences in malaria risk, EIR, and vulnerability across districts and, in some areas, high variability within districts. Important factors contributing to modeled vulnerability patterns included proximity to agricultural fields and water bodies, housing conditions, child malnutrition/stunting, and low education, among others. By decomposing risk into its two components we were able to show that areas with conditions that maximize (minimize) vulnerability to malaria can present high (low) risk if conditions resulting in high (low) entomological inoculation rate prevail. This has crucial policymaking implications. Interventions aimed at reducing vulnerability to malaria could be less effective if adverse conditions that result in an increase in the number of expected bites from infected mosquitoes prevail. Moreover, these conditions vary spatially. Thus, the ideal intervention package should address local conditions that ultimately result in high malaria risk. Web-based spatial decision support tools, such as the WebGIS application presented by Kienberger et al. [61], could be a valuable step towards a more interactive visualization and exploration of these conditions. To increase user confidence in the presented approach the impact of indicator weights on the modeling outputs was evaluated by means of a sensitivity analysis, which revealed that, despite minor differences in the vulnerability surfaces, indicator weights have hardly any impact on modeled malaria risk in the study area. Comparing the three vulnerability surfaces (see Additional file 1: Figure S3, panel 1a to 1c) it becomes obvious that weights based on logistic regression reveal a much smoother result than equal weights or weights based on PCA. This is not surprising since logistic regression captures the contribution of each variable for the outcome in a manner that the two other approaches cannot. It is important to note that areas with differences in malaria vulnerability are mostly the ones with contrasting results for susceptibility and lack of capacity to anticipate and cope. In this case, equal weights are expected to lead to different results when compared to a weighting scheme that considers variable weights for each variable, particularly when higher weights are observed among variables for one particular domain and lower weights for variables in another domain.

This study has some limitations. First, relevant vulnerability indicators, such as acquired immunity to malaria, migration patterns, quality of the health care system, availability of malaria drugs, personal beliefs and behavior, and social networks, among others, were not available in spatially disaggregated format. Nevertheless, the 18 malaria risk factors considered are those often included in malaria modeling exercises, and do provide a good and useful description of malaria risk and vulnerability. Second, also related to data availability, indicators were drawn on data from different years – the oldest being 2002, and the most recent from 2013 (Table 1). Some of the oldest information is likely to have changed in some regions, such as the 2002 data on rural extent. Third, the logistic regression model used to identify relevant risk factors and weights for generating the vulnerability and risk surfaces is a nonspatial model, based on the assumption that the relationships between malaria risk and potential risk factors are stationary [51]. Considering the heterogeneous distribution of most risk factors across the country (Additional file 1: Figure S1), this is likely to be a strong assumption, resulting in a smoother vulnerability surface. Explicit approaches, such as geographically weighted logistic regression, could better capture the presence of spatial effects and indicate areas where some of the variables could have positive, negative, or no effect on the outcome (in nonspatial models some variables can show unexpected effects as a result of significant spatial correlation) [62]. Yet, it is unlikely that the final risk surface would be dramatically different than the one generated here, given the magnitude of the EIR effect.

Strengths of this paper include the systematic modeling framework, the fact that the modeling framework can be easily replicated, and the comprehensive selection of indicators. Upon the availability of updated information, new risk and vulnerability maps can be generated, and allow the assessment of potential changes (improvement or deterioration) that happened across space and over time. These changes, or lack of thereof, could then be contrasted with specific programs implemented by the government (e.g., child health programs, urban planning efforts, and improvement of infrastructure).

## Conclusions

This paper presented a holistic and spatial-explicit approach for assessing malaria risk in Tanzania, taking into account differences in vulnerability and EIR. Multidimensional vulnerability was modeled considering generic and biological susceptibility and lack of resilience. The analysis showed that the root causes of both malaria risk and vulnerability vary considerably across the country. The results presented make three important contributions. First, the risk, hazard (EIR), and vulnerability maps facilitate the prioritization of areas for malaria control. Second, the decomposition of malaria risk in its components, vulnerability domains, and contributing factors provide local evidence of which issues need to be addressed to effectively reduce malaria risk. Lastly, the conceptual framework here presented can be used as a guidance tool for future risk and vulnerability assessments and for monitoring changes over time.

## Additional file

Additional file 1:
**Multicollinearity statistics and indicators.** **Tables S1** to **S4** give the correlation matrix and tables **S5** to **S8** the multicollinearity statistics for all four vulnerability domains (SUS, BIO, C2A, C2C). **Table S9** summarizes the weights used for sensitivity analysis. **Figure S1** shows the spatial patterns, while **Figure S2** displays the normalized values (on a scale from 0 to 100) of the 22 datasets that were included in the analysis. **Figure S3** shows the impact of different weighting schemes on malaria risk and vulnerability. **Figure S4 and S5** present a comparative analysis of malaria vulnerability and malaria risk surfaces using different weighting schemes. **Figure S6** shows the outcomes of the validation of the final risk surface using malaria prevalence data as measured by positive RDTs.
