# Supplementary material for: Mapping malaria risk and vulnerability in the United Republic of Tanzania: a spatial explicit model
Source: Popul Health Metr. 2015 Feb 3;13(1):2. doi: 10.1186/s12963-015-0036-2 (PMC4324856; doi:10.1186/s12963-015-0036-2)
Supplement: Additional file 1: — Multicollinearity statistics and indicators. Tables S1 to S4 give the correlation matrix and tables S5 to S8 the multicollinearity statistics for all four vulnerability domains (SUS, BIO, C2A, C2C). Table S9 summarizes the weights used for sensitivity analysis. Figure S1 shows the spatial patterns, while Figure S2 displays the normalized values (on a scale from 0 to 100) of the 22 datasets that were included in the analysis. Figure S3 shows the impact of different weighting schemes on malaria risk and vulnerability. Figure S4 and S5 present a comparative analysis of malaria vulnerability and malaria risk surfaces using different weighting schemes. Figure S6 shows the outcomes of the validation of the final risk surface using malaria prevalence data as measured by positive RDTs. [file 12963_2015_36_MOESM1_ESM.pdf]

Additional File 1

Table S1. Correlation matrix (Generic susceptibility indicators)

Indicators	SUS_01	SUS_02	SUS_03	SUS_04	SUS_05
SUS_01: Occupation in forestry/fisheries/agriculture (%)	1.000	0.002	-0.040	0.023	0.007
SUS_02: Relative share of agricultural areas (%)	0.002	1.000	-0.006	0.024	0.023
SUS_03: Relative share of water bodies (%)	-0.040	-0.006	1.000	-0.003	-0.065
SUS_04: Number of refugee/IDP camps	0.007	0.024	-0.003	1.000	0.003
SUS_05: Poor housing conditions (%)	0.368	0.023	-0.065	0.003	1.000

SUS = generic susceptibility.

Table S2. Correlation matrix (Biological susceptibility indicators)

Indicators	BIO_01	BIO_02	BIO_03	BIO_04
BIO_01: Children under the age of 5 (%)	1.000	0.358	-0.039	0.030
BIO_02: Women of childbearing age (%)	0.358	1.000	0.103	0.095
BIO_03: Number of HIV-infected persons	-0.039	0.103	1.000	0.547
BIO_04: Number of stunting children under the age of 5	0.030	0.095	0.547	1.000

BIO = biological susceptibility.

Table S3. Correlation matrix (Lack of capacity to anticipate indicators)

Indicators	C2A_01	C2A_02	C2A_03	C2A_04
C2A_01: No/primary education (%)	1.000	0.555	-0.278	-0.010
C2A_02: No phones: landline/mobile (%)	0.555	1.000	-0.282	0.136
C2A_03: Child did not sleep under net last night (%)	-0.278	-0.282	1.000	-0.059
C2A_04: No indoor residual spraying (%)	-0.010	0.136	-0.059	1.000

C2A = lack of capacity to anticipate.

Table S4. Correlation matrix (Lack of capacity to cope indicators)

Indicators	C2C_01	C2C_02	C2C_03	C2C_04
C2C_01:Travel time to closest urban center (minutes)	1.000	-0.067	-0.052	-0.181
C2C_02: No means of transportation: bicycle/motorcycle/ car/truck (%)	-0.067	1.000	-0.224	0.182
C2C_03: No health insurance (%)	-0.052	-0.224	1.000	0.074
C2C_04: Density of health-related projects (km2)	-0.181	0.182	0.074	1.000

C2C = lack of capacity to cope.

Table S5. Multicollinearity statistics (Generic susceptibility indicators)

Indicators	R^2	VIF
SUS_01: Occupation in forestry/fisheries/agriculture (%)	0.183	1.225
SUS_02: Relative share of agricultural areas (%)	0.003	1.003
SUS_03: Relative share of water bodies (%)	0.005	1.005
SUS_04: Number of refugee/IDP camps	0.001	1.001
SUS_05: Poor housing conditions (%)	0.141	1.164

SUS = generic susceptibility; VIF = variance inflation factor.

Table S6. Multicollinearity statistics (Biological susceptibility indicators)

Indicators	R^2	VIF
BIO_01: Children under the age of 5 (%)	0.136	1.157
BIO_02: Women of childbearing age (%)	0.143	1.166
BIO_03: Number of HIV-infected persons	0.308	1.444
BIO_04: Number of stunting children under the age of 5	0.302	1.432

BIO = biological susceptibility; VIF = variance inflation factor.

Table S7. Multicollinearity statistics (Lack of capacity to anticipate indicators)

Indicators	R^2	VIF
C2A_01: No/primary education (%)	0.438	1.779
C2A_02: No phones: landline/mobile (%)	0.353	1.546
C2A_03: Child did not sleep under net last night (%)	0.127	1.145
C2A_04: No indoor residual spraying (%)	0.033	1.034

C2A = lack of capacity to anticipate; VIF = variance inflation factor.

Table S8. Multicollinearity statistics (Lack of capacity to cope indicators)

Indicators	R^2	VIF
C2C_01: Travel time to closest urban center (minutes)	0.036	1.038
C2C_02: No means of transportation: bicycle/motorcycle/ car/truck (%)	0.091	1.101
C2C_03: No health insurance (%)	0.066	1.070
C2C_04: Density of health-related projects (km2)	0.073	1.078

C2C = lack of capacity to cope; VIF = variance inflation factor.

Table S9. Weights used for local sensitivity analysis (based on regression coefficients, PCA, and equal weights)

Indicator name	Weights based on regression[b]	Weights based on PCA	Equal weights
HAZ_01: Entomological inoculation rate (EIR)	0.476	0.074	0.055
SUS_01: Agricultural areas (%)	0.023	0.028	0.055
SUS_02: Density of violent conflicts (km2)	-	-	-
SUS_03: Location of refugee camps	0.003	0.030	0.055
SUS_04: Poor housing conditions (%)	0.022	0.041	0.055
SUS_05: Occupation: forestry/agriculture/fisheries (%)	0.019	0.117	0.055
SUS_06: Rural extent	-	-	-
SUS_07: Water bodies (%)	0.020	0.035	0.055
BIO_01: Children under the age of 5 (%)	0.005	0.059	0.055
BIO_05: Women of childbearing age (%)	0.005	0.051	0.055
BIO_06: Number of HIV infected individuals (15-49 years)	0.054	0.072	0.055
BIO_08: Number of stunting children under 5 years	0.020	0.068	0.055
C2A_01: No/primary education (%)	0.038	0.105	0.055
C2A_02: Does not know how to avoid malaria (%)	-	-	-
C2A_03: No phones (cell/landline)	0.062	0.075	0.055
C2A_04: Child did not sleep under net last night (%)	0.066	0.052	0.055
C2A_05: No indoor residual spraying (%)	0.028	0.033	0.055
C2C_01: Travel time to closest urban center (hours)	0.018	0.016	0.055
C2C_02: No health insurance (%)	0.001	0.044	0.055
C2C_03: No bicycle/motorcycle/car or truck (%)	0.083	0.048	0.055
C2C_04: Density of health-related projects (km2)	0.057	0.056	0.055

[b] For consistency, indicators without weights were removed from the analysis as they were not statistically significant (p-value < 0.05) in the regression model.

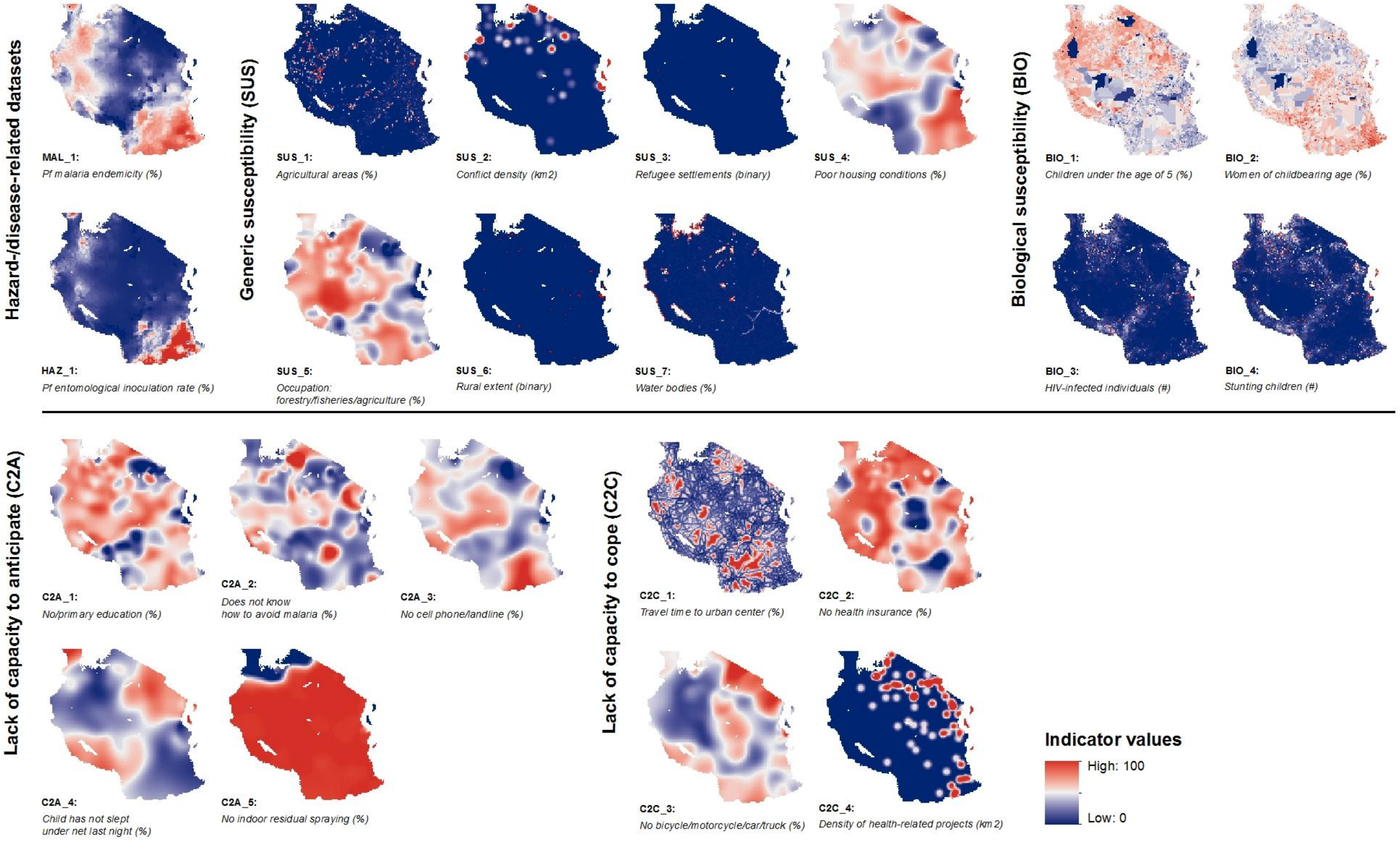

Figure S1. Spatial distribution of indicators included in the analysis
All indicators were normalized, and vary between 0-100.

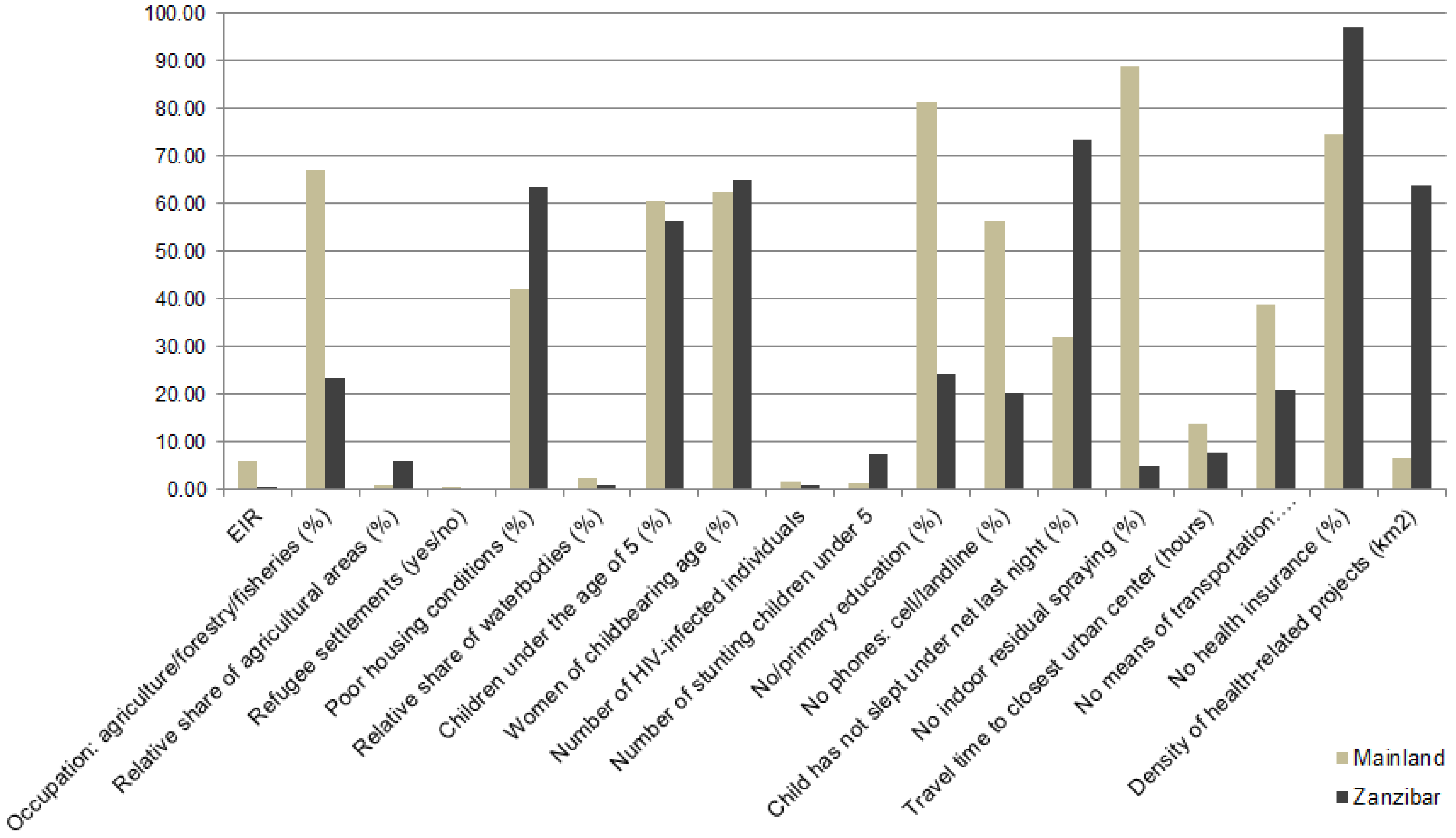

Figure S2. Mean values of final malaria risk factors for Tanzania Mainland and Zanzibar

Figure S2 shows the mean values of the normalized malaria risk factors (incl. hazard/disease and vulnerability indicators) before they were adjusted for their sign for Tanzania Mainland and Zanzibar.

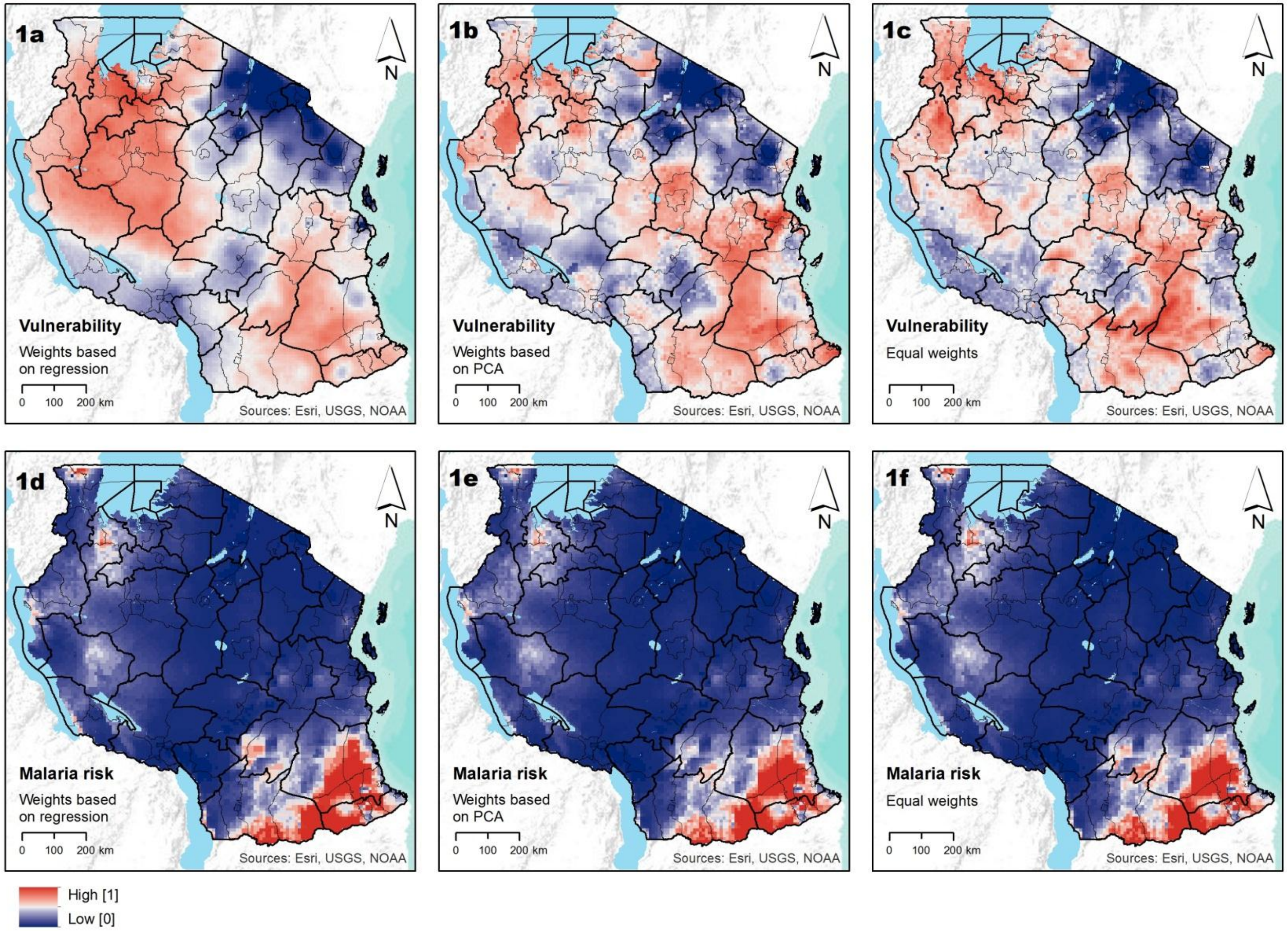

Figure S3. Impact of different weighting schemes on malaria risk and vulnerability

Panels 1a, 1b, and 1c show the vulnerability surface considering weights based on logistic regression, PCA analysis, and equal weights, respectively. Panels 1d, 1e, and 1f show the risk surface considering weights based on logistic regression, PCA analysis, and equal weights, respectively.

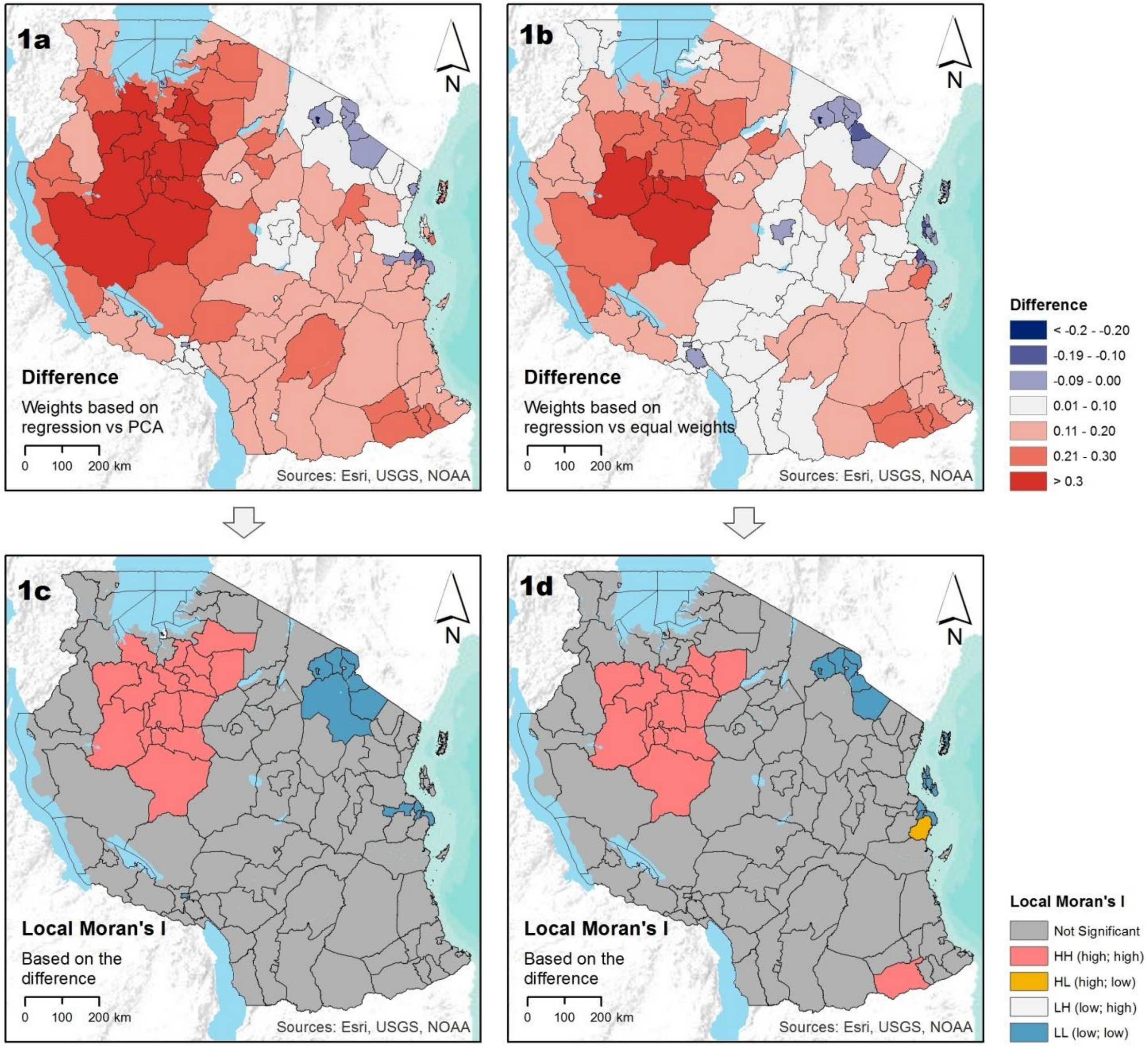

Figure S4. Comparative analysis of vulnerability surfaces using different weighting schemes

Panel 1a shows the difference between vulnerability surfaces based on logistic regression and PCA, while panel 1b shows the difference between vulnerability surfaces based on logistic regression and equal weights. Panels 1c and 1d show the spatial pattern of the differences mapped in panels 1a and 1b, respectively. HH indicates that a district where the vulnerability surface based on logistic regression exhibits significantly higher values than the approach based on PCA is surrounded by other districts characterized by the same pattern. In contrast, LL indicates that a district where the vulnerability map based on logistic regression revealed significantly lower values than the approach based on PCA is surrounded by neighborhoods that show a similar pattern. HL represents areas where the approach based on logistic regression revealed higher vulnerability values that are surrounded by areas where the PCA-based approach revealed lower vulnerability values than the regression approach.

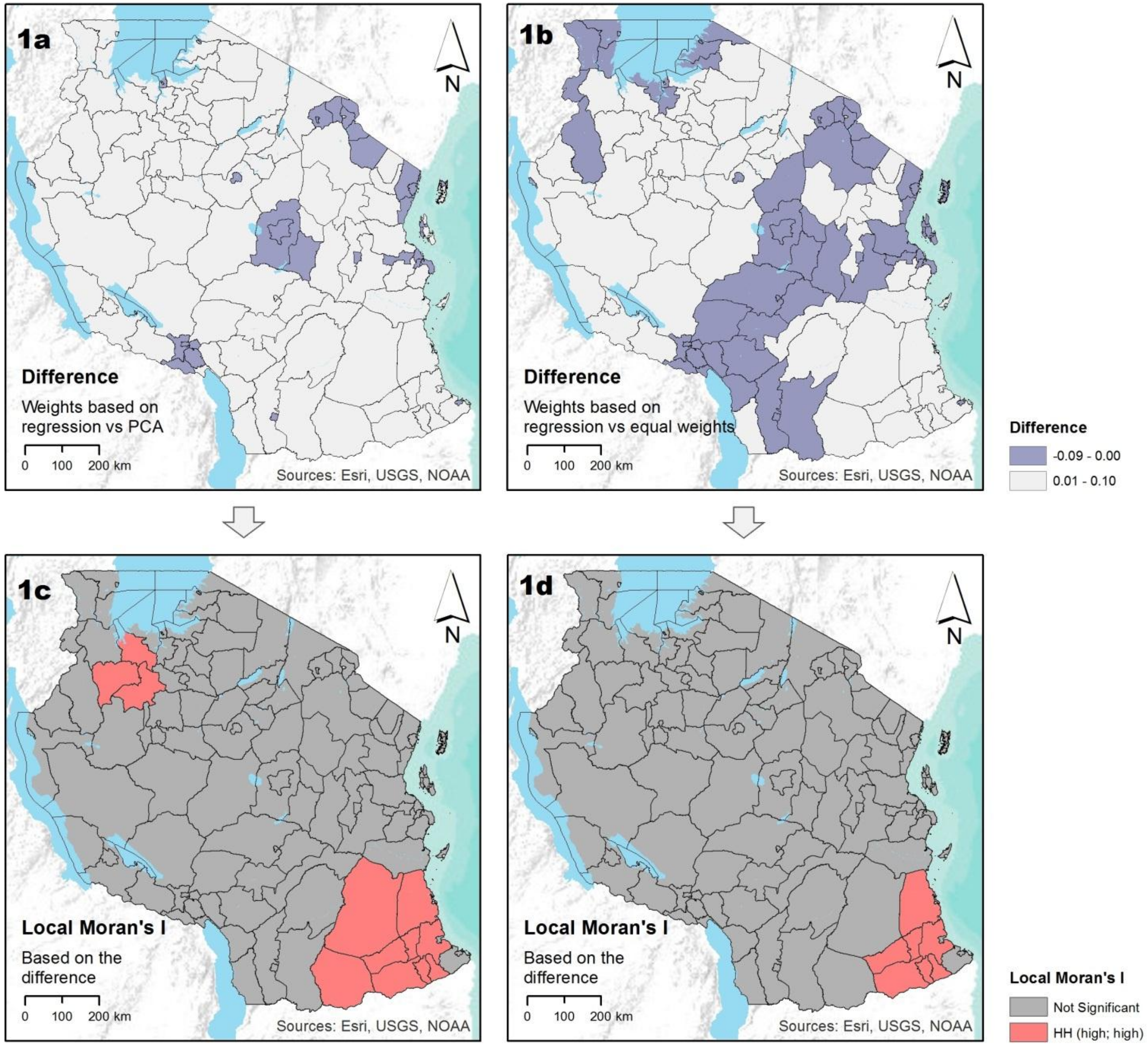

Figure S5. Comparative analysis of malaria risk surfaces using different weighting schemes

Figure S5 (panel 1a) shows the difference between malaria risk based on logistic regression vs. PCA, while panel 1b shows the difference between malaria risk based on logistic regression vs. equal weights. Panels 1c and 1d show the results of the respective cluster analysis based on these differences. HH indicates that a district where the risk surface based on logistic regression exhibits higher values than the approach based on PCA is surrounded by other districts characterized by the same pattern.

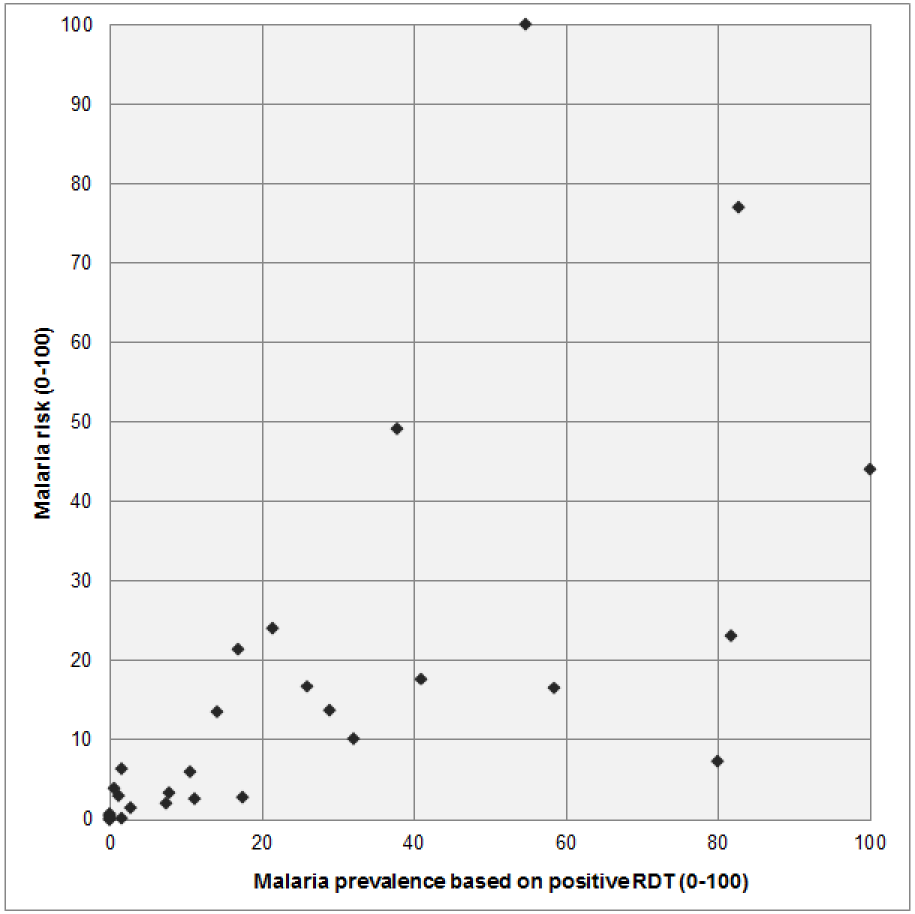

Figure S6. Malaria risk versus malaria prevalence based on positive RDT for the 30 regions of Tanzania
Figure S6 plots the results of the malaria risk assessment versus malaria prevalence based on positive RDTs for the 30 regions of Tanzania.
